# Effect of dietary supplementation of two fiber sources differing on fermentability and hydration capacity on performance, nutrient digestibility and cecal fermentation in broilers from 1 to 42 d of age

**DOI:** 10.1016/j.psj.2024.103957

**Published:** 2024-06-07

**Authors:** A. Rybicka, P. Medel, M.D. Carro, J. García

**Affiliations:** ⁎ Departamento de Producción Agraria, Universidad Politécnica de Madrid, Madrid 28040, Spain; † Innovabiotics, S.L. 28906 Getafe, Madrid, Spain

**Keywords:** fermentation, fructooligosaccharides (FOS), hydration capacity, ileal digestibility, type of fiber

## Abstract

A total of 378 Cobb-500 male broilers were used to evaluate the effects of 2 fiber sources, differing in hydration capacity and fermentability, on gastrointestinal tract development, apparent ileal digestibility and performance from 1 to 42d of age. There were 9 replicates per each of the 3 dietary treatments, all in mash form: a wheat-soybean control (CON) diet, CON diet diluted with 1.5% of wood lignocellulose (LC diet) as a non-fermentable insoluble fiber with high hydration capacity; and CON diluted with 1.5% of a mixture of fibers (ISFC diet) containing both lignified insoluble fiber and a prebiotic soluble fiber fraction from fructooligosaccharides. Additionally, the fermentability of both fiber sources (LC and ISFC) was determined by in vitro using cecal inoculum from broilers fed the experimental diets. Both LC and ISFC treatments impaired by 4% feed conversion ratio only during the first 7d (*P* = 0.003) compared with CON group. In the grower period (21–42d), the ISFC group showed the best growth (*P* = 0.039), and at 42d tended to show the highest body weight (*P* = 0.095). This agrees well with the highest ileal dry matter (*P* = 0.033) and organic matter (*P* = 0.043) digestibility observed in ISFC group and the similar trend observed for ileal protein digestibility (*P* = 0.099) at 42d. Also, at 42 d, absolute and relative (% body weight) digestive tract weights (*P* ≤ 0.041) and empty gizzard weights (*P* ≤ 0.034) were greater for LC and ISFC groups compared to CON. The cecal molar proportion of valeratewas greatest in ISFC group (*P* = 0.039). In vitro gas production was higher for ISFC than for LC substrate when using either a diet-adapted or non-adapted cecal inoculum (*P* < 0.05). These results show the interest in combining IF with prebiotic highly fermentable fiber, such as fructooligosaccharides, in broilers to improve nutrient digestibility and finishing performance.

## INTRODUCTION

The physiological effects of fibrous ingredients on broilers performance depend on different factors, including the characteristics of the basal diet (composition and the fiber levels), intrinsic properties of the fiber source (i.e., solubility, particle size (**PS**), and hydration capacity) and inclusion level, as well as the characteristics of the animals such as the age or specie (Mateos et al., 2012; Berrocoso et al., 2020). An adequate fiber level may promote performance (Zhang et al., 2023), and moderate to high-level supplementation (2.5–7.5%) of insoluble fiber (**IF**) sources to low (4.2–5.5% NDF) fiber diets has been reported to induce several beneficial effects on the gastrointestinal tract (**GIT**), improving barrier function and intestinal health (Jiménez-Moreno et al., 2009; González-Alvarado et al., 2010; Jha et al., 2019). Moderate inclusion level (3.0%) in medium fiber (11.0–11.4% NDF) diets may also improve intestinal immunity, and barrier function and induce beneficial changes in microbial composition in broiler chickens ([Bibr bib0057][Bibr bib0058]). The effect of fiber sources is usually mediated by an increase of the gizzard activity, being especially relevant in fiber sources characterized by coarse PS that lead to more efficacious digestibility of nutrients (Svihus, 2011, 2014). However, finely ground fiber concentrates have attracted research attention for their easier management in the feed mill, absence of mycotoxins, lower inclusion level (10–15 kg/ton) than classical fiber sources, and positive effects on intestinal physiology and growth traits. Previous investigations noticed better nutrient digestibility in broilers by feeding finely than coarse-ground IF such as soyhulls, oat hulls, and cellulose (Jiménez-Moreno et al., 2010; Tejeda and Kim, 2021a). Dietary supplementation of finely ground fiber increased the thickness of the cecal mucus layer, which can prevent aggression by enteric pathogens (Hou et al., 2020), and it may help to maintain the small and large intestine's integrity by strengthening mucosal structure and functions and by increasing the population and diversity of commensal bacteria in the GIT (Jha and Misha, 2022).

Typically, the investigated fiber concentrates are based on wood lignocellulose. However, its high hydration capacity may impair feed intake and growth in young broilers (0–21d), compared with lower hydration capacity fiber sources (Rybicka et al., 2024). Agricultural by-products provide a wide range of lignocellulose-rich sources that properly treated and mixed could be used as alternatives and contribute to a circular economy in the livestock sector. Thus, dietary supplementation of almond hulls improved the AMEn and nitrogen digestibility in laying hens (Wang et al., 2021). Grape by-products are rich in fiber and may also contribute to animal health through its antioxidant activity and scavenging free radicals due to its high polyphenols content (Goñi et al., 2007; Brenes et al., 2008, 2016). Furthermore, many IF sources are normally highly lignified and very low fermentable, and therefore the potential to modulate the microbial intestinal environment is moderate. Low doses of highly fermentable soluble fiber may favorably affect the GIT microbial community, and induce a potential immune response (Shang et al., 2015; Kumar et al., 2019; Tejeda and Kim, 2021b). Fiber sources with prebiotic activity, such as inulin, fructooligosaccharides (**FOS**), xylooligosaccharides, or mannan oligosaccharides, have been observed to promote a beneficial shift in the intestinal microbiota, leading to a decrease of pathogenic species in the cecum and an increase in *Lactobacillus* abundance (Teng and Kim, 2018; Xia et al., 2019). However, to our best knowledge, no previous work has evaluated the potential effects of mixtures of finely ground IF and highly fermentable fibers that would provide different properties and may improve animal health and productivity. We hypothesized that the addition of fiber sources with a moderate HC and partially fermentable may be more beneficial than high HC and non-fermentable for performance. The objective of this trial was therefore to investigate the effects of dietary supplementation of 2 fiber sources, insoluble wood lignocellulose and a mixture of medium-hydration capacity IF originated from different agricultural by-products with a prebiotic fiber source, on performance, GIT development, and nutrient digestibility in broilers from 1 to 42 d of age. Additionally, an *in vitro* fermentation trial was carried out to assess the capacity of the cecal microbiota to ferment both fiber sources as substrates.

## MATERIAL AND METHODS

This study was performed in the experimental facilities of the Universidad Politécnica de Madrid (Spain). Animals were handled according to Spanish guidelines for experimental animal protection (Boletín Oficial del Estado, 2013) and experimental protocols were approved by the Ethics Committee of the Polytechnic University of Madrid (approvement date: 19th of November 2019).

### Husbandry

A total of 378 one-day-old male broilers (Cobb-500) with an initial BW of 38.5 ± 1.02g were obtained from a commercial hatchery. The birds were weighed in groups and distributed according to a completely randomized design into 27 floor pens (150 cm long x 100 cm wide x 50 cm high) equipped with first and second-age feeders and drinkers. All first-age equipment was retired at 7 d. The animals were bedded on pressed straw. The temperature was controlled automatically throughout the trial according to the animal´s age needs (Cobb, 2018). Bird's mortality was recorded daily.

### Diets and Experimental Design

The diets were formulated to meet or exceed the nutritional recommendations for broilers from 1 to 42 d (FEDNA, 2018). There were 3 experimental diets. The control (**CON**) diets consisted of a starter diet (1 to 21 d) based on wheat and soybean meal (**SBM**), containing (as-fed basis) 3,066 kcal AMEn/kg and 1.15 % standardized ileal digestible (**SID**) lysine, and a finishing diet (21– 42 d of age) based on wheat, corn and SBM containing 3,090 kcal AMEn/kg and 1.05% SID lysine (Table 1). The nutritional content of the CON starter diet for moisture, ash, ether extract, and neutral detergent fiber was 10.6, 6.25, 4.48, and 10.2%, respectively, whereas for the CON finishing feed was 10.9, 5.93, 4.17, and 9.94, respectively. The other experimental diets were prepared by diluting the CON diet with 1.5% of 2 different fiber sources: finely ground wood lignocellulose **(LC)** characterized by high hydration capacity and low-fermentability (**LC** diet), or a fiber (**ISFC)** consisting in a mixture of medium-hydration capacity IF sources with a soluble prebiotic fiber fraction (ISFC diet). The wood lignocellulose came from wood pellets (*Pinus spp*., decorticated). The ISFC consisted of a mixture of micronized almond shells, grape skin by-products, olive kernel, wood-lignocellulose, nuts shells, and straw as IF sources, and FOS as soluble-prebiotic source. Both LC and ISFC diets were prepared by mixing the CON diet with the corresponding fiber source for 150 s in a mixer (MMG 316, 250L, Murcia, Spain). In addition, titanium dioxide (TiO2, 0.5%) was included in the finishing diets fed from 38 to 42 d as a marker to determine apparent ileal digestibility. All diets were administered in mash form.

**Table 1:** Ingredient and chemical composition of control diets (%, as-fed basis).

Items	Starter	Finishing
Ingredients, %		
Wheat	59.90	55.13
Soybean meal	32.40	27.92
Corn	0	10.00
Soyben oil	3.17	2.67
Calcium carbonate	1.56	1.74
Monocalcium phosphate	0.96	0.58
L-Lysine	0.39	0.40
DL-Metionine	0.36	0.32
L-Threonine	0.12	0.11
L-Valine	0.09	0.08
Salt	0.31	0.31
Sodium bicarbonate	0.10	0.10
Vitamin and mineral premix^1^	0.30	0.30
PX Maxiban^2^	0.20	0.20
ENP enzyme dry^3^	0.05	0.05
Choline chloride	0.05	0.07
Calculated nutritional content		
AMEn (kcal/kg)	3,066	3,090
SID Lys	1.15	1.05
Moisture	10.6	10.9
Total ash	6.25	5.93
Ether extract	4.48	4.17
Neutral detergent fiber	10.16	9.94

1 Per kg of diet: Vitamin A (Retinyl acetate) (3a672a) 9,000 I.U., Vitamin D3 (Cholecalciferol) (3a671) 3,000 I.U., Vitamin E (all-rac-α-tocopheryl acetate) (3a700) 30 mg, Vitamin K3 (menadione nicotinamide bisulfite) (3a711) 2.5 mg, Vitamin B1 (thiamine mononitrate) (3a821) 2.175 mg, Vitamin B2 (riboflavin) 6 mg, Calcium D-pantothenate (3a841) 10 mg, Vitamin B6 (pyridoxine hydrochloride) (3a831) 2.5 mg, Vitamin B12 (cyanocobalamin) (3a835)15 mg, Niacin (nicotinic acid) (3a314) 40 mg, Folic acid (3a316) 1.5 mg, Biotin (3a880) 0.12 mg, Betaine (Betaine hydrochloride) (3a925) 250 mg, Iron (Iron (II) sulfate, monohydrate) (3b103) 30 mg, Copper (Copper (II) sulfate pentahydrate) (3b405) 8 mg, Manganese (Manganese (II) oxide) (3b502) 80 mg, Zinc (Zinc sulfate, monohydrate) (3b605) 60 mg, Iodine (Coated granulated anhydrous calcium iodate) (3b203) 0.8 mg, Selenium (Sodium selenite) (E8) 0.25 mg, citric acid 0.225 mg, Butylhydroxytoluene (**BHT**) (E321) 0.9 mg, Propyl gallate (E310) 0.075mg, Sepiolite (E562) 3,000 mg, digestibility improvers 6-Phytase EC (3.1.3.26) 667 FYT/g.

2 Per kg of diet: 160 ppm of narasin and 160 ppm of nicarbacin.

3 Per kg of diet: 4a7 Endo-1,4-beta-xylanase EC 3.2.1.8 5,600 TXU, 4a7 Endo-1,4-beta-glucanase EC 3.2.1.4 2,500 TGU.

### Growth Performance

Each treatment was replicated 9 times, and the experimental unit was the pen (14 birds/pen). At d 1, all the birds were weighted in groups of 14, and the individual BW per each pen was calculated. Both, birds BW and feed intake were determined at 7, 21 and 42 d of age, and the data were used to calculate ADFI, ADG and feed conversion ratio (**FCR**) for each period.

### Gastrointestinal Traits

At 7, 21, and 42 d of age, 3 birds per pen (27 birds /treatment) were randomly selected, slaughtered by CO_2_ asphyxiation, and individually weighted. The GIT from the proventriculus to the cloaca was removed and weighed. Then, the different organs (proventriculus, gizzard, and cecum) were separated and weighed individually. The pH of the proventriculus, gizzard, and cecum content was measured using a digital pH meter (model 507, Crison Instruments S.A., Barcelona, Spain), the organs were emptied of digesta, and finally were weighed again to calculate the empty weight.

### Short Chain Fatty Acids Analysis

At 42 d, 3 birds per pen were randomly selected and slaughtered as described before. Then, the cecal content was pooled per pen and immediately frozen (*−*20 °C) until analysis of short-chain fatty acids (**SCFA**) concentration. The mixture of the 3 cecal contents per pen was used as a replicate. There were 9 replicates (pens) per treatment. Sample processing was adapted from Kimiaeitalab et al. (2017). Briefly, samples were defrosted at 4°C, 3 g were weighed, mixed with 5 mL of 0.5 *M* HCl, homogenized, and centrifuged (13,000 *× g*, 15 min, 4 °C). One mL of the supernatant was mixed with 0.5 mL of a deproteinizing solution (20 g metaphosphoric acid and 0.6 g of crotonic acid per L of 0.5 *M* HCl) and left overnight at 4°C. Samples were centrifuged (13,000 *× g*, 15 min, 4 °C) and the supernatant was transferred to chromatography vials. Analysis of SCFA concentrations was performed by gas chromatography using a Shimadzu GC 2010 chromatography (Shimazdu Europa GmbH, Duisburg, Germany) fitted with a TR-FFAP column (30 m × 0.53 mm × 1 µm; Supelco, Madrid, Spain) as described by García-Martínez et al. (2005).

### Apparent Ileal digestibility

From 38 to 42 d, birds were fed the finishing diets containing TiO_2_. On d 42, birds were slaughtered by CO_2_ asphyxia, and ileal digesta was collected from the last third part of the ileum. The contents were gently squeezed into plastic containers, pooled by pen (3 birds/pen), frozen at *−*80 °C, and freeze-dried. Samples of feed and ileal content were ground using a centrifugal mill (Retsch Model Z-I, Stuttgart, Germany) provided with a 1-mm screen, and the AID of dry matter (**DM**), organic matter (**OM)**, CP, and ether extract (**EE**) was determined by using TiO_2_ as a marker. The determination of TiO_2_ in the ileal digesta and diets was performed in accordance with Short et al. (1996). Briefly, 100 mg of freeze-dried sample was ashed at 580°C for 13 h. Ten mL of sulfuric acid (7.4 M) was carefully added to each crucible upon cooling, and samples were then gently boiled for 3 min. After cooling, the solutions were poured into a 100 mL volumetric flask containing 25 mL distilled water through Whatman filter paper (90 mm, WHA1443185). A total of 20 mL of hydrogen peroxide (30%) was added to each flask, and finally, the content was diluted up to 100 mL with distilled water. The absorbance was measured in triplicate using a spectrophotometer (Epoch, BioTek, Santa Clara, CA) at 410 nm***.*** The AID was calculated by the equation as follows:

$$\text{AID}\left[\%\right]=\left[1-\left(\text{Ti}O_{2\text{diet}}\cdot \text{Nutrien}t_{\text{faeces}}\right)/\left(\text{Ti}O_{2\text{faeces}}\cdot {\text{Nutrient}}_{\text{diet}}\right)\right]\cdot 100\%,$$

where TiO_2diet_: insoluble marker content in diet; TiO_2faeces_: insoluble marker content in feces; Nutrient_feces_: nutrient content in feces; Nutrient_diet_: nutrient content in diet. The results were expressed in percentage.

### In Vitro Cecal Fermentation

An in vitro fermentation trial was carried out using the cecal content of 42-d-old birds as inoculum and the 2 fiber sources (LC and ISFC) as substrates. The inoculum from CON birds was considered as non-adapted to fiber sources, whereas that from birds fed either LC or ISFC treatments was considered adapted to the corresponding fiber source. Samples of 200 mg DM of each fiber source were accurately weighed into 60-mL glass vials. A total of 16 birds per dietary treatment were slaughtered by asphyxiation with CO_2_, and the ceca were immediately removed. Within each dietary treatment, the cecal content of 4 birds was pooled to make 4 different inoculums per diet (4 replicates/treatment). Each inoculum was prepared by mixing 1.5 g of cecal content with 100 mL of the culture medium described by Goering and Van Soest (1970). The mixture was homogenized for 20s using a hand blender and strained through a double layer of cheesecloth. Vials were filled up with the mixture using a peristaltic pump (Watson-Marlow 520UIP31; Watson-Marlow Fluid Technology Group, United Kingdom) under continuous flushing with CO_2_, before being sealed with rubber stoppers and incubated at 40°C for 96 h. For each inoculum, 2 vials with each fiber source (LC and ISFC) and 2 vials without substrate (blank) were incubated, making a total of 72 vials (48 with substrate and 24 blanks). The blanks were included to correct the gas production values for the gas produced by the fermentation of the substrates added with the cecal content used as inoculum. Gas production was measured at 2, 7, 12, 48, 72, and 96 h, by using a digital pressure gauge (HD 2,304.0, Delta OHM, Italy) and a plastic syringe. The gas values of the 2 vials for each inoculum and substrate were averaged before statistical analysis.

### Laboratory Analysis

Procedures of the AOAC (2005) were used to determine DM (934.01), ash (942.05), EE (920.39), total dietary fiber (**TDF**, 985.29), and Nitrogen by Dumas (968.06), using a Leco analyzer (model FP-528; Leco Corp., St. Joseph, MI). The CP content was determined by multiplying the N content by 6.25. Neutral detergent fiber (**NDF**), acid detergent fiber (**ADF**), and acid detergent lignin (**ADL**) were determined sequentially using the 25-µm particle retention filter bags (F57; Ankom Technology, New York, NY) by adapting the method of Mertens et al. (2002) and Horwitz et al. (2006). Analysis of NDF was performed using amylase and without any sodium sulphite added, and values were corrected for ash and CP. The soluble fiber content was calculated TDF minus NDF. All analyses were performed in duplicate.

The PS, expressed as geometric mean diameter (**GMD**), and the PS distribution of the two fiber sources, were determined in 100 g samples using a sieve shaker (FTS-0200, Filtra, Badalona, Spain) provided with 5 sieves ranging in mesh from 62 to 1,000 μm (>1,000, 500–1,000, 250–500; 250–105; 105–62; <62 μm; ASAE, 1995). The hydration capacity was estimated by determining the water binding (**WBC**, g/g) and swelling capacities (**SC**, g/mL) by adapting the methods from Slama et al. (2019), Berrocoso et al. (2020) and Priester et al. (2020). Briefly, 0.4 g of each fiber source was hydrated with 10 g of water for 22 h and the mixture was centrifuged (3,100 x *g*, 20 min, Centrifuge, 5810R, Eppendorf, Wesseling-Berzdorf, Germany). The unabsorbed water was weighed, and the WBC was calculated as the difference between the weight of the water added (10 g) and that of the supernatant divided by the initial weight of the sample. The SC was measured using 1 g of sample that was mixed with 20 ml of water under gently stirring and left in a metric cylinder for 22 h. The SC was calculated as the final volume (mL) of the sample divided by the initial sample weight (g).

### Statistical Analysis

The effects of dietary treatments on the measured in vivo parameters were analyzed by one-way analysis of variance (**ANOVA**) with the diet as the main effect. Additionally, orthogonal contrasts were applied to study differences between CON and fiber-supplemented animals (C1: CON vs. LC+ISFC), and between both fiber concentrates (C2: LC vs. ISFC). The pen was an experimental unit for all the statistical analysis, with the only exception of the GIT trait were performed by using 3 birds per pen (n = 27).

*In vitro* gas production data were analyzed for each measurement time and independently for each preplanned comparison. Differences between fiber sources (LC and ISFC) in gas production were tested when the cecal content from CON-fed broilers was used as inoculum (non-adapted inoculum). Potential effects of the experimental diets on gas production were tested independently for each fiber source (LC and ISFC) used as substrate in the *in vitro* trial. For all statistical analyses, effects were considered significant at *P* < 0.05, and trends were considered at *P* < 0.10. When a significant effect of diet was detected, multiple comparisons were carried out by using the Tukey test. Statistical analyses were performed with SPSS (IBM SPSS Statistics for Windows, Version 26.0. Armonk, NY: IBM Corp).

## RESULTS AND DISCUSSION

The fiber composition and physical properties of fiber sources are presented in Table 2. Both LC and ISFC had high amounts of TDF, but the proportions of hemicellulose, cellulose, and lignin were different (16.1, 40.0, 23.4% in LC vs. 26.3, 26.1, 17.9% in ISFC, respectively). Moreover, ISFC contained FOS which would indicate greater fermentability due to higher soluble fiber content (10.8% vs. 16.0 for LC and ISFC, respectively). The analysis of PS confirmed that both products were finely ground but had different PS due to differences in GMD (97 *vs.* 68 μm for LC and ISFC, respectively) and PS distribution. The current methodology was performed due to its common application for the feed PS determination, and previously was used with different fiber sources such as oat hulls, cellulose, pea hulls, rice hulls, sunflower hulls and sugar beet pulp (Jiménez-Moreno et al., 2009; 2010; 2011; 2016; Berrocoso et al., 2020). However, clumping of particles was observed on the top sieves of ISFC sample, which may impact in both particle distribution and GMD, since this method is mostly suitable for spherical and cuboid shapes particles. Also, the FOS addition may have increased the aggregates formation.

**Table 2:** Fiber fractions composition and physicochemical properties of the 2 experimental fiber sources.

	Fiber sources^1^	
Fiber composition (%; as-fed basis)	LC	ISFC
Total dietary fiber (**TDF**)	90.3	86.4
Neutral detergent fiber (**NDF**)	79.5	70.4
Acid detergent fiber (**ADF**)	63.4	44.0
Acid detergent lignin (**ADL**)	23.4	17.9
Hemicellulose^2^	16.1	26.3
Cellulose^3^	40.0	26.1
Soluble dietary fiber^4^	10.8	16.0
Physico-chemical properties		
Geometric mean diameter (**GMD**) (µm)	97	68
Particle size distribution (%)		
>1,000 µm	0	6.67
500–1,000 µm	0	10.8
250–500 µm	1.6	16.8
150–250 µm	26.0	15.7
106–150 µm	39.1	12.0
63–106 µm	21.6	13.1
<63 µm	9.2	24.9
Hydration capacity		
Water binding capacity (WBC) (g/g)	5.8	2.7
Swelling capacity (SC) (g/mL)	8.4	3.0

1 LC: insoluble high-hydration capacity fiber; ISFC: medium-hydration capacity and partially fermentable prebiotic fiber.

2 As NDF – ADF.

3 As ADF – AFL.

4 As TDF – NDF.

It should be highlighted that the Ankom filter bags used in this trial to analyze fiber fractions content of fiber sources were able to retain particles > 25 μm and given the fine particle size of ISFC it is possible that NDF content had been underestimated due to loss of the finer particles (Hall and Mertens, 2023). The water binding and swelling capacities of ISFC were 47 and 36% of those of LC, as expected.

The chemical composition of the experimental diets was close to that expected from feed ingredients composition (Table 3). The GMD of the starter and finishing diets were 652 ± 1.77 μm and 616 ± 1.76 μm, respectively, confirming that the experimental diets were finely ground to avoid the additional effect of PS of other ingredients. The addition of fiber sources produced low impact of the PS of the diets (646 ± 1.78 and 645 ± 1.80 μm in a starter, and 610 ± 1.77 and 610 ± 1.78 μm in a finisher diet, for LC and ISFC, respectively).

**Table 3:** Chemical composition (%, as-fed basis) and geometric mean diameter (GMD) of starter and finishing experimental diets^1^.

	Starter			Finishing		
Item	CON	LC	ISFC	CON	LC	ISFC
Organic matter	84.3	85.0	85.2	84.3	84.6	84.7
Dry matter	90.6	90.5	90.7	90.1	89.9	90.1
Crude protein	22.1	22.4	22.6	21.0	20.5	20.2
Ether extract	4.29	4.21	4.40	4.72	4.31	4.33
Neutral detergent fiber	11.4	13.8	13.7	11.8	13.0	12.7
Acid detergent fiber	3.35	4.28	4.39	3.61	4.56	4.42
Acid detergent lignin	0.81	1.04	1.29	0.72	1.00	1.32
GMD^2^ (µm)	652	646	645	616	610	610

1 CON: standard non-fiber supplemented diet; LC: CON diet diluted with 1.5% of insoluble high hydration properties fiber source; ISFC: CON diet diluted with 1.5% of insoluble medium hydration properties and partially fermentable prebiotic fiber source.

2 Geometric mean diameter.

### Growth Performance and Nutrient Digestibility

The mortality rate was low (2.9% on average) and was not related to dietary treatments (data not shown). The influence of dietary treatments on broiler performance is shown in Table 4. From 1 to 7 d of age, dietary supplementation with both fiber sources impaired FCR by 4% (*P* = 0.003) but this effect disappeared thereafter. The increase of FCR between 1 and 7 d was most probably a result of the energetic and nutritional dilution produced by 1.5% of fiber inclusion. In this case, several adaptation mechanisms may take place to maintain the growth rate. The modern highly selected birds can compensate the dietary reduced nutrient concentrations due to fiber addition by increasing feed intake (Jha and Misha, 2022), although ADFI differences in our trial were not significant (*P* = 0.60). Changes in FCR disappeared with time, and FCR values were similar in all treatments from 7 d onwards and in the whole period (*P* ≥ 0.18). Different fiber sources were reported to have a negative effect on FCR in young chicks. Dietary lignocellulose inclusion at 1 and 2% increased FCR in broilers at 7 d, although 2% supplementation resulted in a significant recover of FCR at 35 d (Sozcu, 2019). Sugar beet pulp or rice hulls supplemented at 3% to the basal diet impaired FCR from 1 to 14 d in chicks but had no effect in the overall period (1-42 d) (Saadatmand et al., 2019). These findings support that dietary fiber inclusion produces digestive tract modifications in young broilers.

**Table 4:** Influence of fiber sources differing in hydration capacity and fermentability on broiler's performance from 1 to 42 d^1^.

		Dietary treatments^1^			SEM^2^	*P-*value		
Time	Item	CON	LC	ISFC		Treatment	C1^3^	C2^4^
1–7 d	BW at 7 d (g)	161	159	158	1.96	0.59	0.31	0.89
	ADG (g/d)	17.5	17.2	17.2	0.27	0.58	0.30	0.95
	ADFI (g/d)	17.0	17.5	17.5	0.24	0.60	0.32	0.89
	FCR (g/g)	0.981^a^	1.017^b^	1.022^b^	0.0096	0.012	0.003	0.75
7–21 d	BW at 21 d (g)	857	838	841	10.3	0.38	0.17	0.86
	ADG (g/d)	49.8	48.6	48.8	0.70	0.46	0.22	0.88
	ADFI (g/d)	65.4	64.5	65.1	1.28	0.88	0.72	0.75
	FCR (g/g)	1.316	1.327	1.336	0.0250	0.86	0.63	0.81
1–21 d	ADG (g/d)	38.9	38.1	38.2	0.48	0.37	0.17	0.84
	ADFI (g/d)	49.3	48.8	49.3	0.83	0.91	0.78	0.73
	FCR (g/g)	1.267	1.283	1.290	0.0217	0.76	0.48	0.83
21–42 d	BW at 42 d (g)	3035	3046	3136	34.1	0.095	0.195	0.076
	ADG (g/d)	103.7^b^	105.2^ab^	109.3^a^	1.49	0.039	0.068	0.063
	ADFI (g/d)	163.2	164.5	167.9	1.95	0.24	0.23	0.23
	FCR (g/g)	1.575	1.564	1.536	0.0150	0.18	0.18	0.20
1–42 d	ADG (g/d)	71.4	71.6	73.7	0.81	0.094	0.19	0.075
	ADFI (g/d)	106.3	106.6	108.6	1.23	0.39	0.39	0.28
	FCR (g/g)	1.490	1.490	1.472	0.0117	0.45	0.50	0.29

a,b Means in the same row not sharing a common letter differ (*P* < 0.05).

1 CON: standard non-fiber supplemented diet; LC: CON diet diluted with 1.5% of insoluble high hydration properties fiber source; ISFC: CON diet diluted with 1.5% of insoluble medium hydration properties and partially fermentable prebiotic fiber source.

2 SEM, n=9.

3 C1: CON vs. LC+ISFC.

4 C2: LC vs. ISFC.

In the finishing period (21– 42 d of age), ISFC-fed broilers had greater ADG than CON animals, presenting LC intermediate values (*P* = 0.039). This effect remained as a trend in the overall period (*P* = 0.094). Feeding ISFC also tended to result in greater BW than CON at 42 d (*P* = 0.076) but no effect of dietary treatments on ADFI was detected in any period (*P* ≥ 0.23). In previous investigations performed using a standard corn-SBM diet, the administration of different micronized IF sources (cellulose or lignocellulose) at low levels (0.25–0.75%) increased ADG of broilers between 1 and 42 d (Sarikhan et al., 2010; Rezaei et al., 2011; Makivic et al., 2018). In contrast, higher levels (1-2%) of lignocellulose inclusion had limited effects on animal growth in the finishing period (Kheravi et al., 2017). Besides, different supplementation levels of prebiotic compounds as FOS (0.15%) or inulin (0.12–4%) have been reported to increase broilers’ growth in the finishing period (Yang et al., 2016; Xia et al., 2019). Likewise, chicory root powder supplemented at 0.1 to 1.0% improved performance in male chickens at 42 d (Khoobani et al., 2019; Gurram et al., 2021). These positive effects were probably due to an increase of beneficial microbiota in the GIT, due to the prebiotic activities of the supplements, which contributed to gut health.

Birds fed ISFC showed 4% higher AID of both DM (*P* = 0.013) and OM (*P* = 0.016) compared with LC-fed birds, although values were similar to those in CON group. Feeding ISFC tended to increase AID of CP in comparison to CON, presenting LC-fed birds intermediate values (*P* = 0.099; Table 5). Values of AID of CP were positively correlated with ADG from 1 to 42 d (*r* = 0.998; *P* = 0.036; n = 3) and with BW at 42 d (*r* = 0.997; *P* = 0.050, n = 3). Nutrient digestibility is normally depressed by high dietary fiber levels, but moderate amounts of fiber have been considered as one of the alternatives proposed to improve nutrient digestibility and performance in broilers (Khempaka et al., 2009; Tejeda and Kim, 2020; Jha and Misha, 2022). Finely ground fiber sources have shown a positive influence on nutrient digestibility in broilers (Jiménez Moreno et al., 2010; Tejeda and Kim, 2021a). Greater AID in ISFC than LC is in agreement with our previous observation that finely ground almond shells were retained more time in the small intestine and may lead to more efficacious digestibility than LC, hypothesizing a stimulation of reverse peristalsis and digestive enzyme secretion (González-Alvarado et al., 2007; Rybicka et al., 2024). The highly fermentable fraction of FOS may be partially fermented before reaching the cecum, resulting in better AID of DM and OM compared to LC. Highly fermentable oligosaccharides can improve nutrient digestibility due to beneficial modifications of gastrointestinal microbiota and increased immune function (Huang et al., 2005; Yadav and Jha, 2019). Dietary addition of FOS at 0.05% improved DM, crude fiber, CP, and EE digestibility in broilers, which was associated with an increased activity of digestive enzymes in the small intestine (Saleh et al., 2014). Similarly, Xu et al. (2003) reported that the administration of 0.4% of FOS enhanced protease, amylase, and lipase secretion and improved the morphology of intestinal mucosa leading to increased growth of broilers. In addition, intestinal morphology improvements may be associated with better nutrient digestibility, and several studies have reported changes in intestinal morphology by fiber supplementation. In the present trial, the intestinal morphology could not be reported due to autolysis of samples. However, in a previous study (Rybicka et al., 2024) we observed that LC supplementation at 1.5% produced no effect on intestinal morphology in 21-d broilers, whereas in the current experiment, the same level of LC supplementation had a negative effect on AID of DM and OM. Therefore, the positive effects of ISFC on digestibility and broiler growth may be associated with the FOS fraction or its interaction with IF. These results indicate that despite of the very fine PS of both fiber sources, including a highly fermentable fraction (such as FOS) may be relevant to improve nutrient digestibility, as fermentability of high-lignified IF sources cannot be improved by reducing their PS.

**Table 5:** Influence of fiber sources differing in hydration capacity and fermentability on apparent ileal digestibility (%) of dry matter (**DM**), organic matter (**OM**), crude protein (**CP**) and ether extract (**EE**) in broilers at 42 d of age^1^.

	Dietary treatments^1^			SEM^2^	*P-*value		
Item	CON	LC	ISFC		Treatment	C1^3^	C2^4^
DM	83.5^ab^	82.7^b^	86.1^a^	0.90	0.033	0.41	0.013
OM	85.7^ab^	85.0^b^	87.8^a^	0.78	0.043	0.49	0.016
CP	87.8	87.9	89.9	0.73	0.099	0.25	0.066
EE	91.0	91.2	92.1	0.77	0.55	0.50	0.39

a,b Means in the same row not sharing a common letter differ (*P* < 0.05).

1 CON: standard non-fiber supplemented diet; LC: CON diet diluted with 1.5% of insoluble high hydration properties fiber source; ISFC: CON diet diluted with 1.5% of insoluble medium hydration properties and partially fermentable prebiotic fiber source.

2 SEM, n=9.

3 C1: CON vs. LC+ISFC.

4 C2: LC vs. ISFC.

### GIT Traits

No effects on GIT traits were observed at 7 or 21d (*P* ≥ 0.17). At 42 d, the full weight of both the whole digestive tract (*P* = 0.019) and gizzard (*P* = 0.030) were increased by fiber inclusion (Table 6). Compared with CON birds, fiber supplementation increased the full weight of gizzard (*P* = 0.030) and tended to increase the full weight of gizzard + proventriculus (*P* = 0.054). When the weights of the digestive organs were expressed relative to broilers BW, the full digestive tract remained heavier in LC-fed birds than in CON birds, whereas the ISFC group showed intermediate values (*P* = 0.041; Table 7). The heavier weight of the whole digestive tract was probably associated with high bulking properties of LC. Particles of IF can incorporate water into their matrix and swell during their passage through the GIT, resulting in a bulkier digesta (González-Alvarado et al., 2007). Indeed, the greater capacity to retain water of LC compared with ISFC (Table 2) may have a direct impact on the full weight of the GIT.

**Table 6:** Influence of fiber sources differing in hydration capacity and fermentability on BW and gastrointestinal traits of broilers at 42 d^1^.

	Dietary treatments^1^			SEM^2^	*P-*value		
Item	CON	LC	ISFC		Treatment	C1^3^	C2^4^
BW (g)	3,016	3,089	3,137	64.2	0.41	0.22	0.61
Full weight (g)							
Whole digestive tract	233b	260a	249ab	7.3	0.038	0.019	0.30
Gizzard	62.3	72.1	68.4	2.95	0.065	0.030	0.38
Proventriculus	13.4	17.8	14.4	2.34	0.39	0.35	0.31
Cecum	21.2	22.3	24.9	1.17	0.083	0.11	0.12
Gizzard+Proventiculus	75.7	89.9	82.8	4.43	0.085	0.054	0.26
SICR^5^	136	148	142	5.3	0.30	0.19	0.40
Empty weight (g)							
Gizzard	34.8b	37.3ab	39.9a	0.98	0.002	0.002	0.070
Proventriculus	9.8	10.6	10.6	0.56	0.49	0.23	0.96
Gizzard+Proventiculus	44.5b	47.9ab	50.5a	1.31	0.005	0.003	0.15
Content weight (g)							
Gizzard	27.5	34.8	28.5	2.37	0.069	0.16	0.065
Proventriculus	3.71	3.25	3.79	1.872	0.32	0.43	0.19
pH							
Gizzard	3.12ab	2.81b	3.29a	0.119	0.021	0.64	0.006
Proventriculus	3.07	3.23	3.25	0.230	0.83	0.55	0.94
Cecum	6.36	6.47	6.54	0.081	0.27	0.14	0.53

Means in the same row not sharing a common letter differ (*P* < 0.05).

1 CON: standard non-fiber supplemented diet; LC: CON diet diluted with 1.5% of insoluble high hydration properties fiber source; ISFC: CON diet diluted with 1.5% of insoluble medium hydration properties and partially fermentable prebiotic fiber source.

2 SEM, n = 27.

3 C1: CON vs. LC+ISFC.

4 C2: LC vs. ISFC.

5 SICR: weight of full small intestine, colon and rectum calculated by difference.

**Table 7:** Influence of fiber sources differing in hydration capacity and fermentability on gastrointestinal tract development relative to BW of broilers at 42 d^1^**.**

	Dietary treatments^1^			SEM^2^	*P*-value		
Item	CON	LC	ISFC		Treatment	C1^3^	C2^4^
Full weight (% BW)							
Whole digestive tract	7.75b	8.41a	7.99ab	0.174	0.032	0.041	0.093
Gizzard	2.07	2.34	2.20	0.096	0.16	0.10	0.32
Proventriculus	0.44	0.58	0.45	0.079	0.40	0.44	0.26
Cecum	0.70	0.72	0.80	0.036	0.13	0.20	0.12
Gizzard+Proventiculus	2.51	2.91	2.65	0.145	0.15	0.13	0.21
SICR^5^	4.54	4.77	4.53	0.125	0.31	0.46	0.18
Empty weight (% BW)							
Gizzard	1.16b	1.21ab	1.28a	0.034	0.038	0.034	0.15
Proventriculus	0.32	0.34	0.34	0.016	0.71	0.42	0.85
Gizzard+Proventiculus	1.48b	1.55ab	1.62a	0.039	0.049	0.030	24
Content weight (% BW)							
Gizzard	0.92	1.12	0.92	0.081	0.10	0.27	0.06
Proventriculus	0.12	0.24	0.11	0.062	0.31	0.47	0.18

Means in the same row not sharing a common letter differ (*P* < 0.05).

1 CON: standard non-fiber supplemented diet; LC: CON diet diluted with 1.5% of insoluble high hydration properties fiber source; ISFC: CON diet diluted with 1.5% of insoluble medium hydration properties and partially fermentable prebiotic fiber source.

2 SEM, n = 27.

3 C1: CON vs. LC+ISFC.

4 C2: LC vs. ISFC.

5 SICR: weight of full small intestine, colon and rectum calculated by difference.

On the opposite, the empty weight of the gizzard was greater in ISFC than in CON group, expressed both in absolute and relative numbers, with LC broilers having an intermediate value (*P* < 0.05). Proventriculus and gizzard are usually considered a functional unit taking part in the digestion process, due to the bidirectional digesta flow between both organs that regulates its flow throughout the GIT (Jiménez-Moreno et al., 2019; Lierman et al., 2019). The enhancement effect of gizzard development produced by ISFC was unexpected, due to its low PS and hydration capacity. Generally, a lack of effect, or even a reduction of gizzard development, were reported by dietary inclusion of finely ground fiber, despite some studies not indicating if full or empty gizzard weights were given (Rezaei et al., 2011; Makiavic et al., 2018). However, a greater gizzard was observed in 28-d Japanese quails due to the dietary addition of 1.5% of micronized cellulose (Rezaei et al., 2018). In fact, it was suggested that dietary supplementation of modified cassava pulp at 1 or 1.5% induced thickening of the gizzard muscular wall due to the hardness of its IF (Okrathok and Khempaka, 2020). This may suggest that there are more factors involved in the gizzard development, than only the PS. For example, in this trial all animals had straw bedding that is a common practice in commercial conditions. Thus, a potential additional source of coarse fiber, cannot be discarded which might have some influence on gizzard development, although no apparent presence of straw or feathers were observed in the digesta.

At 42 d, ISFC-fed birds had greater gizzard pH than those fed LC but similar to CON animals (*P* = 0.021). These results agree well with those of Sadeghi et al. (2015), who observed greater gizzard pH in broilers supplemented with a 50:50 mixture of IF and soluble fiber from rice hulls and sugar beet pulp, than in those fed only IF from rice hulls. Other studies have supplemented the diet with LC, but the results are inconclusive. In broilers, Makiavic et al. (2018) reported a gizzard content acidification by LC supplementation at 0.4 and 0.6% with no effect on gizzard weight, whereas Sozcu (2019) observed no effect on gizzard pH by lignocellulose inclusion at 0.5, 1 or 2% of the total diet. These inconsistent results suggest that for finely ground fiber sources, the effect on the gizzard pH could be affected by additional factors, such as the inclusion level, bird's age, diet composition and structure, and buffer capacity of fiber sources. Normally, the increase in proventriculus and gizzard weight is associated with a reduction of pH, due to higher HCl secretion that was commonly reported for diets containing structural components, such as coarse fibers or coarse grains (Svihus, 2011; Mateos et al., 2012; Okrathok and Khempaka, 2020). However, in the present study the feed was finely ground, and no pH reduction was observed. Despite of higher values of gizzard's pH in ISFC birds, no negative effects were detected on performance or nutrient digestibility.

### Cecal Fermentation

Both, total SCFA and acetate concentrations in the cecum were decreased (*P* = 0.048 and 0.040, respectively) with fiber sources addition (Table 8). These results support the limited cecal fermentation of highly lignified IF sources in poultry (Bautil et al., 2023). Results of the current trial are in line with our previous study that showed no effect of dietary supplementation of LC and other IF sources on cecal total SCFA concentration in 21-d broilers. Previous studies from other groups reported that LC is slightly fermentable in the cecum of swine and turkey (Youssef and Kamphus, 2018), and had a low capacity to modulate cecal SCFA production (Zeitz et al., 2019) and cecal pH (Bogusławska-Tryk et al., 2015). Also, supplementing higher doses (2 and 4%) of LC had no effect on cecal concentrations of SCFA in chickens (Hou et al., 2020).

**Table 8:** The influence of insoluble fiber concentrates differing in hydration capacity and fermentability on the cecal short chain fatty acids (SCFA) concentration in broilers at 42 d^1^**.**

	Dietary treatments^1^			SEM^2^	*P-*value		
Item	CON	LC	ISFC		Treatment	C1^3^	C2^4^
SCFA concentration (µmol/g of cecal content)							
Acetate	92.8	76.0	73.9	6.77	0.11	0.040	0.83
Propionate	13.9	11.3	11.2	1.59	0.39	0.17	0.97
Butyrate	20.3	17.5	16.6	1.75	0.32	0.15	0.70
Isobutyrate	1.07	1.34	1.04	0.11	0.73	0.82	0.44
Valerate	1.46	1.41	1.49	0.12	0.19	0.95	0.64
Isovalerate	1.01	1.11	1.04	0.09	0.73	0.73	0.99
Total SCFA	134.0	114.1	108.5	8.97	0.13	0.048	0.66
Molar proportion of individual SCFA (mol/100 mol)							
Acetate	69.3	66.5	67.6	1.64	0.48	0.27	0.64
Propionate	10.4	9.9	10.0	0.95	0.91	0.67	0.90
Butyrate	15.3	15.5	16.0	1.36	0.94	0.80	0.81
Isobutyrate	0.84	1.2	0.96	0.12	0.085	0.17	0.36
Valerate	1.1b	1.2ab	1.4a	0.10	0.039	0.037	0.13
Isovalerate	0.81	1.045	1.00	0.11	0.28	0.17	0.91

Means in the same row not sharing a common letter differ (*P* < 0.05).

1 CON: standard non-fiber supplemented diet; LC: CON diet diluted with 1.5% of insoluble high hydration properties fiber source; ISFC: CON diet diluted with 1.5% of insoluble medium hydration properties and partially fermentable prebiotic fiber source.

2 SEM, n=9.

3 C1: CON vs. LC+ISFC.

4 C2: LC vs. ISFC.

The question of whether prebiotic sources are able to improve cecal SCFA concentrations in broilers still remains unresolved (Worawang et al., 2022). Feeding FOS during either the whole rearing period or the finishing phase of broilers did not modify cecal concentrations of total SCFA, acetate, propionate, or butyrate (Cao et al., 2005; Ao and Choct, 2013). A metanalysis carried out by Worawong et al. (2022) showed that prebiotic supplementation increased cecal concentrations of propionate and butyrate, with no effects on concentrations of total SCFA and acetate. These results suggest that these easily fermentable prebiotic fiber sources might partially disappear before reaching the cecum. However, prebiotics are widely known to modify the GIT microbiota and to stimulate the growth of cecal beneficial microorganisms (Shang et al., 2018; Teng and Kim, 2018; Kumar et al., 2019; Xia et al., 2019). For that reason, ISFC was designed as a mixture of fibers to maintain the properties of IF, due to its physical effects on GIT development, and a highly fermentable source in form of FOS was included. In the current study, only the molar proportion of valerate in the cecum was modified by fiber inclusion, with greater values in ISFC-fed broilers compared with control ones, presenting LC intermediate values (*P* = 0.039). This is well in accordance with the greater valerate concentration in the cecal content of 42-d broilers fed FOS (4 g/kg) reported by Cao et al. (2005), which also selectively promoted the growth of favorable microbes and inhibited phenol, ethylphenol, cresol, and indole production. Higher levels of valerate were associated with beneficial modification of the microbiota by *L. acidophilus* supplementation in broilers (Li et al., 2017).

A trend of higher weight of cecum was observed in broilers supplemented with ISFC in comparison to CON *(P* = 0.083), but cecal pH was not modified (*P* = 0.27; Table 6). Cecum weight is presumably influenced by PS and the amount of fermentable substrate, which should be fine enough to enter in the cecum (Röhe and Zentek, 2021). In the current study, both fiber sources were finely ground, thus allowing their pass into the cecum. In contrast to our results, the inclusion of 1.5% finely ground almond shell, an inert source with low hydration capacity, tended to decrease the weight of cecum in 21-d broilers (Rybicka et al., 2024). Therefore, the positive effects on cecal weight observed in the current study might be related to the FOS content of ISFC and its high fermentability, which may stimulate the growth of GIT microbiota affecting also the cecum due to peristaltic and antiperistaltic movements (Wedegaertner, 2021).

**Figure 1:**
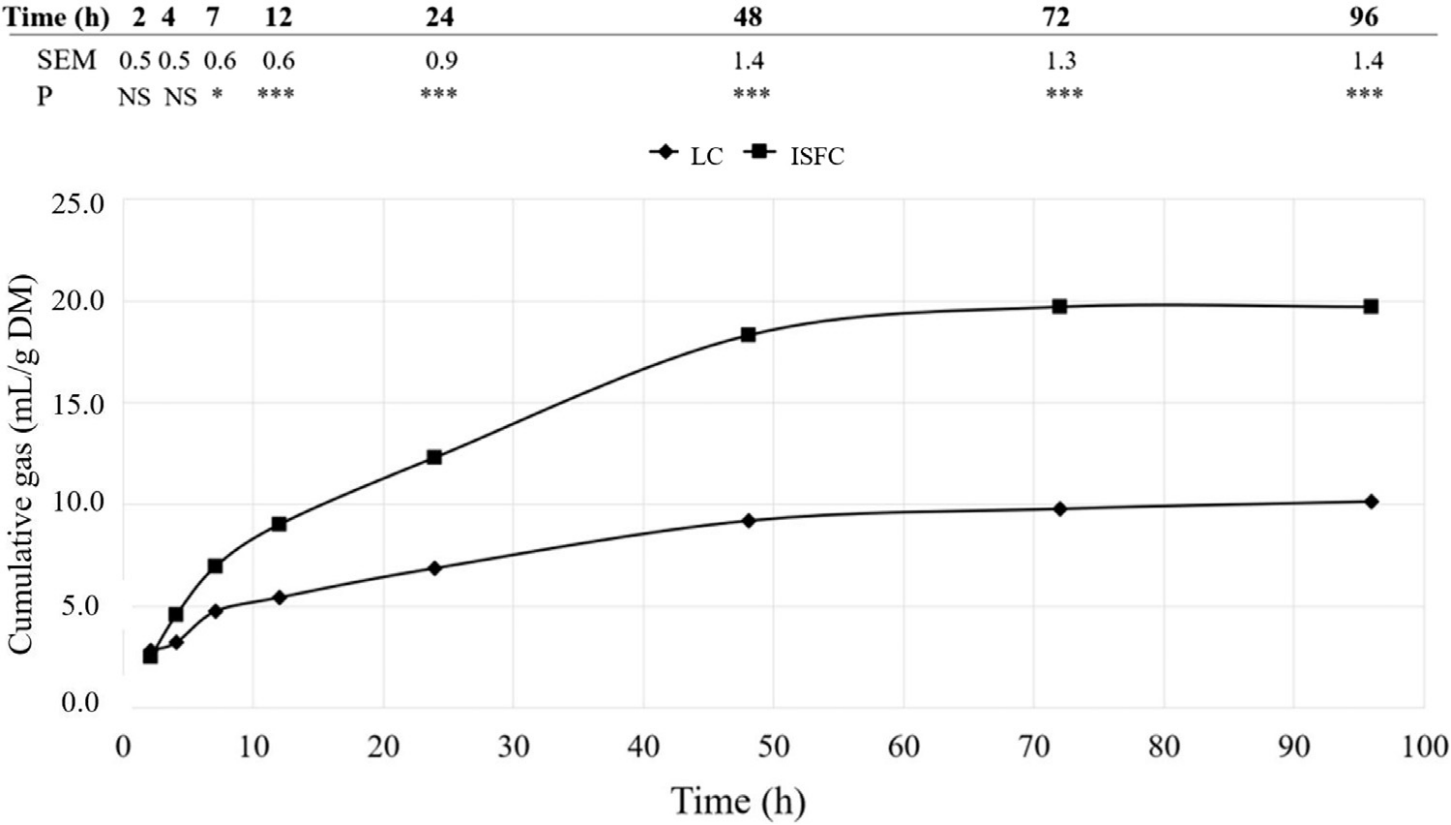
Cumulative gas production curves (mL/gDM) of LC (insoluble and high hydration properties fiber) and ISFC (insoluble and medium hydration properties with partially fermentable prebiotic fiber) substrates after their incubation with cecal content from 42-d birds fed a control diet based on cereals and soybean meal. Four different inocula were used, and each inocula was a pooled cecal content from 4 birds fed the control diet. Values in the Table indicate the SEM and *P* value of the ANOVA analyzing potential differences in gas production at each measurement time. NS= *P* > 0.10; t = 0.10 < *P* < 0.05; * *P* < 0.05; *** *P* < 0.001.

As shown in Figure 1, gas production from ISFC started slightly earlier and had greater values from 7 h of incubation onwards in comparison to LC substrate (*P* < 0.050). The lower content in ADL and greater content in total soluble fiber of ISFC (17.9% and 16.0%, respectively) than in LC (23.4% and 10.8%) can help to explain the greater gas production from ISFC, as gas production is positively related to the OM fermented (Menke and Steingass, 1988). These results are, in general, in good agreement with our previous study in 21-d broilers (Rybicka et al., 2024), confirming the low fermentability of LC and that it does not seem to change with broilers aging.

**Figure 2:**
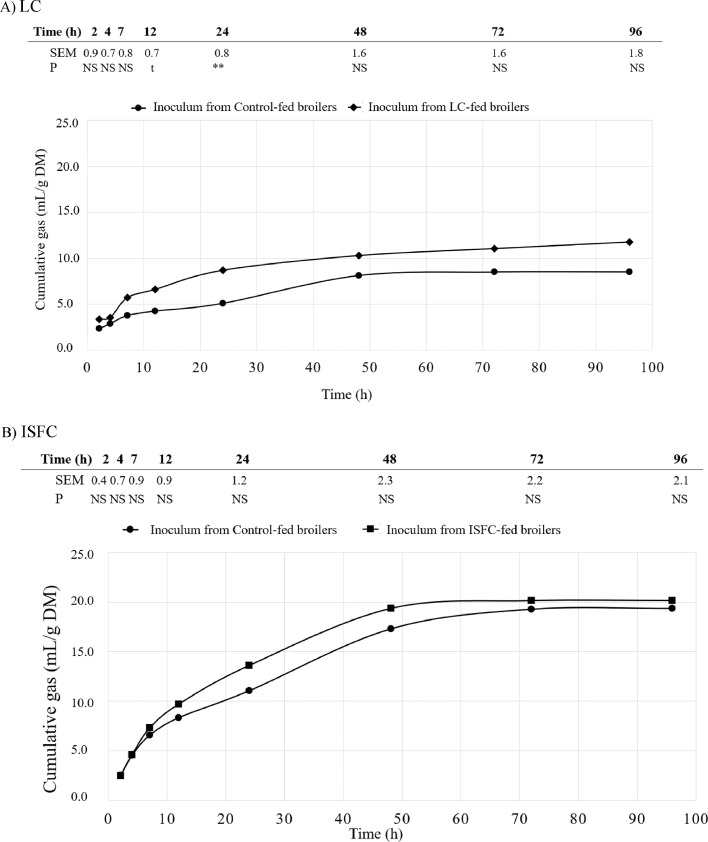
Cumulative gas production curves (mL/gDM) of A) insoluble and high hydration capacity fiber substrate (LC), and B) insoluble and medium hydration capacity with partially fermentable prebiotic fiber substrate (ISFC) when they were fermented either using cecal inoculum from 42-d birds fed a diet including the incubated fiber source (adapted microbiota) or a control diet without LC and ISFC (CON; non-adapted microbiota). Four different inoculums were used for each experimental dietary treatment, and each inocula was a pooled cecal content from 4 birds fed the same diet. Tables indicate the SEM and *P* value of the ANOVA analyzing potential differences in gas production values at each measurement time. Significance was declared at P < 0.05, whereas P < 0.10 values were considered as a trend. NS= *P* > 0.10; t = 0.10 < *P* < 0.05; * *P* < 0.05.

The gas production curves for LC and ISFC substrates when using cecal inoculum from broilers fed CON diet (without exposition to fiber sources) and LC or ISFC diets (with previous exposition to the substrate in the diet) are presented in Figures 2A and 2B. Only small differences between control and substrate-adapted inoculum were observed for LC substrate. The inoculum from LC-fed broilers tended to produce more gas at 12 h (*P* = 0.063) and produced more gas at 24 h (*P* = 0.017) than the inoculum from CON-fed broilers, but differences disappeared thereafter. In contrast, there were no differences (*P* > 0.10) between CON and ISFC inoculums in the amount of gas produced from ISCF fermentation at any incubation time. Therefore, the capacity to ferment fiber did not seem to increase by 42-d exposition to the substrate. Values of gas production were in accordance with those previously observed in 21-d old broilers (Rybicka et al., 2024).

It is generally accepted that the ceca are the main fermentation chambers in the avian GIT and had the highest bacterial density in broilers (Rehman et al., 2007; Wedegaertner, 2021). The FOS used as a fermentable fiber source are not digested in the small intestine (Hajati and Rezaei, 2010), but are selectively and fast fermented by *Lactobacillus, Enterococcus,* and *Pediococcus* spp. strains that were also isolated in other parts of the GIT including the crop (Greppi et al., 2020; Reuben et al., 2019). Despite of the higher fermentability of ISFC determined in vitro (as indicated by the relatively high gas production) compared with LC substrate, the low *in vivo* effects observed on cecal SCFA concentrations and pH may be related to low amounts of ISFC reaching the cecum due to partial fermentation in previous sections of GIT. More studies should be carried out with different types of fiber to characterize the fiber fermentative capacity in the cecum of broilers.

## CONCLUSIONS

The results of the current study show that low levels of dietary supplementation of a mixture of insoluble fiber with prebiotic soluble fiber may be more beneficent for broilers performance in the finishing phase, due to its fermentative patterns and greater apparent ileal digestibility of OM and DM than the non-fermentable insoluble source. However, the mechanism of action remains unclear due to confusing effects on gizzard pH and cecal fermentation. Further investigation would be needed to clarify all the mechanisms involved in the observed response.

## References

[bib0001] American Society of Agricultural Engineers (1995). ASAE Standard.

[bib0002] Ao Z., Choct M. (2013). Oligosaccharides affect performance and gut development of broiler chickens. Asian-Australasian J. Anim. Sci..

[bib0003] AOAC (2005).

[bib0004] Bautil A., Bedford M.R., Buyse J., Courtin C.M. (2023). Reduced-particle size wheat bran and endoxylanase supplementation in broiler feed affect arabinoxylan hydrolysis and fermentation with broiler age differently. Anim. Nutr..

[bib0005] Berrocoso J.D., García-Ruiz A., Page G., Jaworski N.W. (2020). The effect of added oat hulls or sugar beet pulp to diets containing rapidly or slowly digestible protein sources on broiler growth performance from 0 to 36 days of age. Poult. Sci..

[bib0006] Bogusławska-Tryk M., Szymeczko R., Piotrowska A., Burlikowska K., Śliżewska K. (2015). Ileal and cecal microbial population and short-chain fatty acid profile in broiler chickens fed diets supplemented with lignocellulose. Pak. Vet. J..

[bib0007] Boletín Oficial del Estado (2013). Royal Decree 53/2013 of February 1st on the protection of animals used for experimentation or other scientific purposes. BOE n°.

[bib0008] Brenes A., Viveros A., Goñi I., Centeno C., Sáyago-Ayerdy S.G., Arija I., Saura-Calixto F. (2008). Effect of grape pomace concentrate and vitamin E on digestibility of polyphenols and antioxidant activity in chickens. Poult. Sci..

[bib0009] Brenes A., Viveros A., Chamorro S., Arija I. (2016). Use of polyphenol-rich grape by-products in monogastric nutrition. A review. Anim. Feed Sci. Technol..

[bib0010] Cao B.H., Karasawa Y., Guo Y.M. (2005). Effects of green tea polyphenols and fructo-oligosaccharides in semi-purified diets on broilers’ performance and caecal microflora and their metabolites. Asian-Aust. J. Anim. Sci..

[bib0011] Cobb (2018).

[bib0012] FEDNA (Fundación Española para el Desarrollo de la Nutrición Animal). 2018. Necesidades Nutricionales en Avicultura: Normas FEDNA (2ª edición). R. Lázaro, and G. G. Mateos, Madrid, Spain.

[bib0013] García-Martínez R., Ranilla M.J., Tejido M.L., Carro M.D. (2005). Effects of disodium fumarate on in vitro rumen microbial growth, methane production and fermentation of diets differing in their forage:concentrate ratio. Br. J. Nutr..

[bib0014] Goering H.K., Van Soest P.J. (1970).

[bib0015] Goñi I., Brenes A., Centeno C., Viveros A., Saura-Calixto F., Rebolé A., Arija I., Estevéz R. (2007). Effect of dietary grape pomace and vitamin E on growth performance, nutrient digestibility, and susceptibility to meat lipid oxidation in chickens. Poult. Sci..

[bib0016] González-Alvarado J.M., Jiménez-Moreno E., Lázaro R., Mateos G.G. (2007). Effect of type of cereal, heat processing of the cereal, and inclusion of fiber in the diet on productive performance and digestive traits of broilers. Poult. Sci..

[bib0017] González-Alvarado J.M., Jiménez-Moreno E., González-Sánchez D., Lázaro R., Mateos G.G. (2010). Effect of inclusion of oat hulls and sugar beet pulp in the diet on productive performance and digestive traits of broilers from 1 to 42 days of age. Anim. Feed Sci. Technol..

[bib0018] Greppi A., Asare P.T., Schwab C., Zemp N., Stephan R., Lacroix C. (2020). Isolation and comparative genomic analysis of reuterin-producing Lactobacillus reuteri from poultry gastrointestinal tract. Front Microbiol.

[bib0019] Gurram S., Chinni P.V., Vijaya L.K., Raju M.V.L.N., Swathi B. (2021). Supplementation of chicory root powder as an alternative to antibiotic growth promoter on gut pH, gut microflora and gut histomorphometery of male broilers. Plos one.

[bib0020] Hajati H., Rezaei M. (2010). The application of prebiotics in poultry production. Int. J. Poult. Sci.

[bib0021] Hall M.B., Mertens D.R. (2023). Comparison of alternative neutral detergent fiber methods to the AOAC definitive method. J. Dairy Sci..

[bib0022] Horwitz W. (2006).

[bib0023] Hou L., Sun B., Yang Y. (2020). Effects of added dietary fiber and rearing system on the gut microbial diversity and gut health of chickens. Animals.

[bib0024] Huang R.L., Yin Y.L., Wu G.Y., Zhang Y.G., Li T.J., Li L.L., Li M.X., Tang Z.R., Zhang J.J., Wang B., He J.H., Nie X.Z. (2005). Effect of dietary oligochitosan supplementation on ileal digestibility of nutrients and performance in broilers. Poult. Sci..

[bib0025] Jha R., Fouhse J.M., Tiwari U.P., Li L., Willing B.P. (2019). Dietary fiber and intestinal health of monogastric animals. Front. Vet. Sci..

[bib0026] Jha R., Misha P. (2022). Dietary fiber in poultry nutrition and their effects on nutrient utilization, performance, gut health, and on the environment: a review. Anim. Sci. Biotechnol..

[bib0027] Jiménez-Moreno E., González-Alvarado J.M., González-Serrano A., Lázaro R., Mateos G.G. (2009). Effect of dietary fiber and fat on performance and digestive traits of broilers from one to twenty-one days of age. Poult. Sci..

[bib0028] Jiménez-Moreno E., González-Alvarado J.M., González-Sánchez D., Lázaro R., Mateos G.G. (2010). Effects of type and particle size of dietary fiber on growth performance and digestive traits of broilers from 1 to 21 days of age. Poult. Sci..

[bib0029] Jiménez-Moreno E., Chamorro S., Frikha M., Safaa H.M., Lázaro R., Mateos G.G. (2011). Effects of increasing levels of pea hulls in the diet on productive performance, development of the gastrointestinal tract, and nutrient retention of broilers from one to eighteen days of age. Anim. Feed Sci. Technol..

[bib0030] Jiménez-Moreno E., De Coca-Sinova A., González-Alvarado J.M., Mateos G.G. (2016). Inclusion of insoluble fiber sources in mash or pellet diets for young broilers. Effects on growth performance and water intake. Poult. Sci..

[bib0031] Jiménez-Moreno E., González-Alvarado J.M., de Coca-Sinova A., Lázaro R., Cámara L., Mateos G.G. (2019). Insoluble fiber sources in mash or pellets diets for young broilers. Effects on gastrointestinal tract development and nutrient digestibility. Poult. Sci..

[bib0032] Khempaka S., Molee W., Guillaume M. (2009). Dried cassava pulp as an alternative feedstuff for broilers: Effect on growth performance, carcass traits, digestive organs, and nutrient digestibility. J. Appl. Poult. Res..

[bib0033] Kheravii S.K., Swick R.A., Choct M., Wu S.B. (2017). Coarse particle inclusion and lignocellulose-rich fiber addition in feed benefit performance and health of broiler chickens. Poult. Sci..

[bib0034] Khoobani M., Hasheminezhad S.H., Javandel F., Nosrati M., Seidavi A., Kadim I.T., Laudadio V., Tufarelli V. (2019). Effects of dietary chicory (*Chicorium intybus* l.) and probiotic blend as natural feed additives on performance traits, blood biochemistry, and gut microbiota of broiler chickens. Antibiotics.

[bib0035] Kimiaeitalab M.V., Mirzaie S., Jiménez-Moreno E., Cámara L., Mateos G.G. (2017). A comparative study on the effects of dietary sunflower hulls on growth performance and digestive tract traits of broilers and pullets fed a pullet diet from 0 to 21 days of age. Poult. Sci..

[bib0036] Kumar S., Shang Y., Kim W.K. (2019). Insight into dynamics of gut microbial community of broilers fed with fructooligosaccharides supplemented low calcium and phosphorus diets. Front. Vet. Sci..

[bib0037] Li Z., Wang W., Liu D., Guo Y. (2017). Effects of Lactobacillus acidophilus on gut microbiota composition in broilers challenged with Clostridium perfringens. PLoS ONE.

[bib0038] Liermann W., Frahm J., Berk A., Danicke S. (2019). Investigations of relationships between alterations of the gastrointestinal tract caused by feeding variously processed feedstuffs and blood and immunological traits of broilers. Poult. Sci..

[bib0039] Makivic L., Glisic M., Boskovic M., Djordjevic J., Markovic R., Baltic M., Sefer D. (2018). Performances, ileal and cecal microbial populations and histological characteristics in broilers fed diets supplemented with lignocellulose. Kafkas Univ. Vet. Fak. Derg..

[bib0040] Mateos G.G., Jiménez-Moreno E., Serrano M.P., Lázaro R. (2012). Poultry response to high levels of dietary fiber sources varying in physical and chemical characteristics. J. Appl. Poult. Res..

[bib0041] Menke K.H., Steingass H. (1988). Estimation of energetic feed value obtained from chemical analysis and in vitro gas production. Anim. Res. Dev..

[bib0042] Mertens D.R. (2002). Gravimetric determination of amylase-treated neutral detergent fiber in feeds with refluxing in beakers or crucibles: Collaborative study. J. AOAC Int..

[bib0043] Okrathok S., Khempaka S. (2020). Modified-dietary fiber from cassava pulp reduces abdominal fat and meat cholesterol contents without affecting growth performance of broiler chickens. J. Appl. Poult. Res..

[bib0044] Priester M., Visscher C., Fels M., Rohn K., Dusel G. (2020). Fiber supply for breeding sows and its effects on social behaviour in group-housed sows and performance during lactation. Porc. Health Manag..

[bib0045] Rehman H.U., Vahjen W., Awad W.A., Zentek J. (2007). Indigenous bacteria and bacterial metabolic products in the gastrointestinal tract of broiler chickens. Archives Anim. Nutr..

[bib0046] Reuben R.C., Roy P.C., Sarkar S.L., Alam R.U., Jahid I.K. (2019). Isolation, characterization, and assessment of lactic acid bacteria toward their selection as poultry probiotics. BMC Microbiol.

[bib0047] Rezaei M., Karimi Torshizi M.A., Rouzbehan Y. (2011). The influence of different levels of micronized insoluble fiber on broiler performance and litter moisture. Poult. Sci..

[bib0048] Rezaei M., Karimi Torshizi M.A., Wall H., Ivarsson E. (2018). Body growth, intestinal morphology and microflora of quail on diets supplemented with micronised wheat fibre. Br. Poult. Sci..

[bib0049] Röhe I., Zentek J. (2021). Lignocellulose as an insoluble fiber source in poultry nutrition: a review. J. Anim. Sci. Biotechnol..

[bib0050] Rybicka A., del Pozo R., Carro M.D., García J. (2024). Effect of type of fiber and its physicochemical properties on performance, digestive transit time, and cecal fermentation in broilers from 1 to 23 d of age. Poult. Sci..

[bib0051] Saadatmand N., Toghyani M., Gheisari A. (2019). Effects of dietary fiber and threonine on performance, intestinal morphology and immune responses in broiler chickens. Anim. Nutr..

[bib0052] Sadeghi A., Toghyani M., Gheisari A. (2015). Effect of various fiber types and choice feeding of fiber on performance, gut development, humoral immunity, and fiber preference in broiler chicks. Poult. Sci..

[bib0053] Saleh A.A., Amber K., El-Magd M.A., Atta M.S., Mohammed A.A., Ragab M.M., El-Kader H.A. (2014). Integrative effects of feeding *Aspergillus awamori* and fructooligosaccharide on growth performance and digestibility in broilers: Promotion muscle protein metabolism. Biomed Res. Int..

[bib0054] Sarikhan M., Shahryar H.A., Gholizadeh B., Hosseinzadeh M.H., Beheshti B., Mahmoodnejad A. (2010). Effects of insoluble fiber on growth performance, carcass traits and ileum morphological parameters on broiler chick males. Int. J. Agric. Biol..

[bib0055] Shang Y., Regassa A., Kim J.H., Kim W.K. (2015). The effect of dietary fructooligosaccharide supplementation on growth performance, intestinal morphology, and immune responses in broiler chickens challenged with *Salmonella Enteritidis* lipopolysaccharides. Poult. Sci..

[bib0056] Shang Y., Kumar S., Thippareddi H., Kim W.K. (2018). Effect of dietary fructooligosaccharide (FOS) supplementation on ileal microbiota in broiler chickens. Poult. Sci..

[bib0057] Shang Q.H., Liu S.J., He T.F., Liu H.S., Mahfuz S., Ma X.K., Piao X.S. (2020). Effects of wheat bran in comparison to antibiotics on growth performance, intestinal immunity, barrier function, and microbial composition in broiler chickens. Poult. Sci..

[bib0058] Shang Q., Wu D., Liu H., Mahfuz S., Piao X. (2020). The impact of wheat bran on the morphology and physiology of the gastrointestinal tract in broiler chickens. Animals.

[bib0059] Short F.J., Gorton P., Wiseman J., Boorman K.N. (1996). Determination of titanium dioxide added as an marker in chicken digestibility studies. Anim. Feed. Sci. Technol..

[bib0060] Slama J., Schedle K., Wurzer G.K., Gierus M. (2019). Physicochemical properties to support fibre characterization in monogastric animal nutrition. J. Sci. Food Agric..

[bib0061] Sozcu A. (2019). Growth performance, pH value of gizzard, hepatic enzyme activity, immunologic indicators, intestinal histomorphology, and cecal microflora of broilers fed diets supplemented with processed lignocellulose. Poult. Sci..

[bib0062] Svihus B. (2011). Reviews The gizzard: function, influence of diet structure and effects on nutrient availability. Poult. Sci..

[bib0063] Svihus B. (2014). Function of the digestive system. J. Appl. Poult. Res..

[bib0064] Tejeda O.J., Kim W.K. (2020). The effects of cellulose and soybean hulls as sources of dietary fiber on the growth performance, organ growth, gut histomorphology, and nutrient digestibility of broiler chickens. Poult. Sci..

[bib0065] Tejeda O.J., Kim W.K. (2021). Effects of fiber type, particle size, and inclusion level on the growth performance, digestive organ growth, intestinal morphology, intestinal viscosity, and gene expression of broilers. Poult. Sci..

[bib0066] Tejeda O.J., Kim W.K. (2021). Role of dietary fiber in poultry nutrition. Animals.

[bib0067] Teng P.Y., Kim W.K. (2018). Review: Roles of prebiotics in intestinal ecosystem of broilers. Front. Vet. Sci..

[bib0068] Wang J., Kong F., Kim W.K. (2021). Effect of almond hulls on the performance, egg quality, nutrient digestibility, and body composition of laying hens. Poult. Sci..

[bib0069] Wedegaertner O.A. (2021).

[bib0070] Worawong K., Nasri T., Siripornadulsil W., Sukon P. (2022). Effects of prebiotic supplementation on the concentration of short-chain fatty acids in the ceca of broiler chickens: a meta-analysis of controlled trials. Anim. Feed Sci. Techn..

[bib0071] Xia Y., Kong J., Zhang G., Zhang X., Seviour R., Kong Y. (2019). Effects of dietary inulin supplementation on the composition and dynamics of cecal microbiota and growth-related parameters in broiler chickens. Poult. Sci..

[bib0072] Xu Z.R., Hu C.H., Xia M.S., Zhan X.A., Wang M.Q. (2003). Effects of dietary fructooligosaccharide on digestive enzyme activities, intestinal microflora and morphology of male broilers. Poult. Sci..

[bib0073] Yadav S., Jha R. (2019). Strategies to modulate the intestinal microbiota and their effects on nutrient utilization, performance, and health of poultry. J. Anim. Sci. Biotechn..

[bib0074] Yang G.Q., Yin Y., Liu H.Y., Liu G.H. (2016). Effects of dietary oligosaccharide supplementation on growth performance, concentrations of the major odor-causing compounds in excreta, and the cecal microflora of broilers. Poult. Sci..

[bib0075] Youssef I.M.I., Kamphues J. (2018). Fermentation of lignocellulose ingredients in vivo and in vitro via using fecal and caecal inoculums of monogastric animals (swine/turkeys). J. Basic Appl. Sci..

[bib0076] Zhang C., Hao E., Chen X., Huang C., Liu G., Chen H., Wang D., Shi L., Xuan F., Chang D., Chen Y. (2023). Dietary fiber level improve growth performance, nutrient digestibility, immune and intestinal morphology of broilers from day 22 to 42. Animals.

[bib0077] Zeitz J.O., Neufeld K., Potthast C., Kroismayr A., Most E., Eder K. (2019). Effects of dietary supplementation of the lignocelluloses FibreCell and OptiCell on performance, expression of inflammation-related genes and the gut microbiome of broilers. Poult. Sci..

